# Fabrication of 2D-MoSe_2_ incorporated NiO Nanorods modified electrode for selective detection of glucose in serum samples

**DOI:** 10.1038/s41598-021-92620-2

**Published:** 2021-06-24

**Authors:** Gayathri Jeevanandham, Kumaran Vediappan, Zeid A. ALOthman, Tariq Altalhi, Ashok K. Sundramoorthy

**Affiliations:** 1 Department of Chemistry, SRM Institute of Science and Technology, Tamil Nadu, Kattankulathur, 603203 India; 2 Chemistry Department, College of Science, King Saud University, P. O. Box 2455, Riyadh, 11451 Saudi Arabia; 3 Department of Chemistry, College of Science, Taif University, P.O. Box 11099, Taif, 21944 Saudi Arabia

**Keywords:** Nanoscience and technology, Nanoscale materials

## Abstract

Layered molybdenum diselenide (MoSe_2_) nanosheets were formed by the weak Van der Waals forces of attraction between Se and Mo atoms. MoSe_2_ has a larger space between the adjacent layers and smaller band gaps in the range of 0.85 to ~ 1.6 eV. In this study, MoSe_2_ nanosheets decorated nickel oxide (NiO) nanorods have been synthesized by hydrothermal method using sodium molybdate and selenium metal powder. NiO/MoSe_2_ composite formation was confirmed by powder X-ray diffraction analysis. In addition, the presence of MoSe_2_ nanosheets on NiO nanorods were confirmed by field emission scanning electron microscopy, high-resolution transmission electron microscopy and X-ray photoelectron spectroscopy. The Nyquist plots of NiO/MoSe_2_ coated glassy carbon electrode (GCE) was indicated that it had lower charge transfer resistance compared to NiO/GCE and MoSe_2_/GCE. Furthermore, as-prepared NiO/MoSe_2_/GCE was used to detect glucose in alkaline solution by cyclic voltammetry and amperometry techniques. The NiO/MoSe_2_/GCE was exhibited a linear response for the oxidation of glucose from 50 µM to 15.5 mM (R^2^ = 0.9842) at 0.5 V by amperometry. The sensor response time and the limit of detection were found to be 2 s and 0.6 µM for glucose. Moreover, selectivity of the NiO/MoSe_2_ sensor was tested in the presence of common interferent molecules such as hydrogen peroxide, fructose, lactose, ascorbic acid, uric acid, and dopamine. It was found that NiO/MoSe_2_/GCE did not respond to these interfering biomolecules. In addition, NiO/MoSe_2_/GCE had shown high stability, reproducibility and repeatability. Finally, the practical application of the sensor was demonstrated by detecting glucose in human blood serum with the acceptable recovery.

## Introduction

The layered structures of transition metal dichalcogenides (TMDs) such as molybdenum diselenide (MoSe_2_), molybdenum disulphide (MoS_2_), tungsten disulphide (WS_2_) and tungsten diselenide (WSe_2_) have been actively investigated due to their attractive physical and chemical properties. Specifically, MoSe_2_ has a strong interlayered covalent bonds and each layers are sandwiched together by a weaker Van der Waals force of attraction. MoSe_2_ is a semiconductor with the bandgap in the range of 0.85 to ~ 1.6 eV and exhibited high catalytic activity, surface area and conductivity^1^. Compared to MoS_2_, MoSe_2_ had exhibited higher electrical conductivity^2^ and electrocatalytic activity due to its high metallic nature and electrocatalytically active unsaturated edges of Se^3^. Recently, MoSe_2_ had been exploited in various applications, specifically, to enhance the electrocatalytic activity in oxygen reduction reaction (ORR), hydrogen evolution reaction (HER)^4^, supercapacitors^5^, photocatalysis^6^ and sensors^7^. To obtain single-layers of MoSe_2_, various methods have been developed such as electrochemical exfoliation^8^, liquid phase exfoliation^9^, chemical vapor deposition^10^, hydrothermal methods^11^, etc. Zhang et al*.* fabricated MoSe_2_/NiSe_2_ nanowires on carbon fibers which had shown high electrocatalytic activity for the hydrogen evolution reaction^12^. *Harpeness* et al. synthesized MoSe_2_ nanorods with the lengths of 45 to 55 nm by microwave-assisted reaction between Mo(CO)_6_ and Se^13^. Recently, MoSe_2_ based composites such as Ru/MoSe_2_^14^, Rh/MoSe_2_^15^, Co/MoSe_2_^16^_,_ Mn/MoSe_2_^17^_,_ Nb/MoSe_2_^18^, Pd/MoSe_2_^19^_,_ Sr/MoSe_2_^20^ and Er/MoSe_2_^21^ have been reported for various applications which include gas sensors, solar cells^22^, sodium-ion^23^ and lithium-ion batteries^24^. The electrocatalytic activity of MoSe_2_ could be improved further by (i) increasing the active sites by cation doping or substitution and (ii) by making composite with highly conducting carbon materials that can serve as a supporting catalyst to enhance the electrocatalytic activity^20^.

Recently transition metal oxides such as nickel oxide (NiO)^25^, zinc oxide^26^, iron oxide (Fe_3_O_4_)^27^, cerium oxide (CeO_2_)^28^ and tin oxide (SnO_2_)^29^ have been used as active catalytic materials to construct glucose sensors because these metal oxides can be easily synthesized with high stability at low cost. Among the various metal oxides, NiO had shown well-defined redox activity in alkaline (NaOH) solution due to the stable Ni^2+^/Ni^3+^ redox reaction. NiO is a *p*-type semiconductor with the bandgap in the range of 3.6 to 4.0 eV^30^. In order to further explore the electrocatalytic properties and applications of NiO, it had been synthesized in various shapes such as nanoparticles^25^, nanosheets^31^, nanoflowers^32^, nanofibers^33^, nanoplates^34^, hollow sphere ^35^ and nanoflakes^36^. Specifically, NiO had shown high electro-catalytic activity towards glucose. So, NiO-based nanocomposites such as NiO/graphene nanosheets^37^, NiO/MWCNTs^38^, Ni/NiO-rGO^39^ and Ni/carbon^40^ have been used to construct non-enzymatic glucose sensors. However, these reported sensors had shown some limitations such as utilization of expensive nanomaterials and reagents, requirement of higher working potential, short detection range, higher limit of detection (LOD), etc.

In order to increase the sensitivity and selectivity of the electrochemical sensors, various hybrid materials have been synthesized and used to construct glucose sensors by enzymatic and non-enzymatic methods. Although, the enzyme-based glucose biosensors have shown high selectivity and sensitivity, they are susceptible to environmental variations such as pH and temperature which could deteriorate the device performance due to the denaturing of glucose oxidase^41^. To overcome such problems and reduce the cost of the sensor devices, non-enzymatic glucose sensors have been considered for further developments. At the same time, it is anticipated that the demand for simple and accurate glucose monitoring devices is growing due to the surge in the number of diabetic patients.

In this study, we have reported synthesis of NiO nanorods in the presence of MoSe_2_ nanosheets. As-prepared NiO/MoSe_2_ nanocomposite was characterized by PXRD, FE-SEM, HR-TEM, XPS and EDX analysis. In addition, electrochemical and electrocatalytic properties of NiO/MoSe_2_ nanocomposite modified glassy carbon electrode (NiO/MoSe_2_/GCE) were studied by cyclic voltammetry, amperometry and electrochemical impedance spectroscopy (EIS). Interestingly, NiO/MoSe_2_/GCE had shown an enhanced electro-catalytic activity towards glucose oxidation at 0.5 V in 0.1 M NaOH. Using amperometry, a linear response was obtained for glucose oxidation from 50 µM to 15.5 mM. The common interferent molecules such as hydrogen peroxide (H_2_O_2_), fructose, lactose, uric acid (UA), dopamine (DA) and ascorbic acid (AA) were tested in the presence of glucose on NiO/MoSe_2_/GCE. Finally, accurate detection of glucose in human blood serum was demonstrated by using NiO/MoSe_2_/GCE as a non-enzymatic sensor.

## Results and discussion

### PXRD analysis

**Figure 1:**
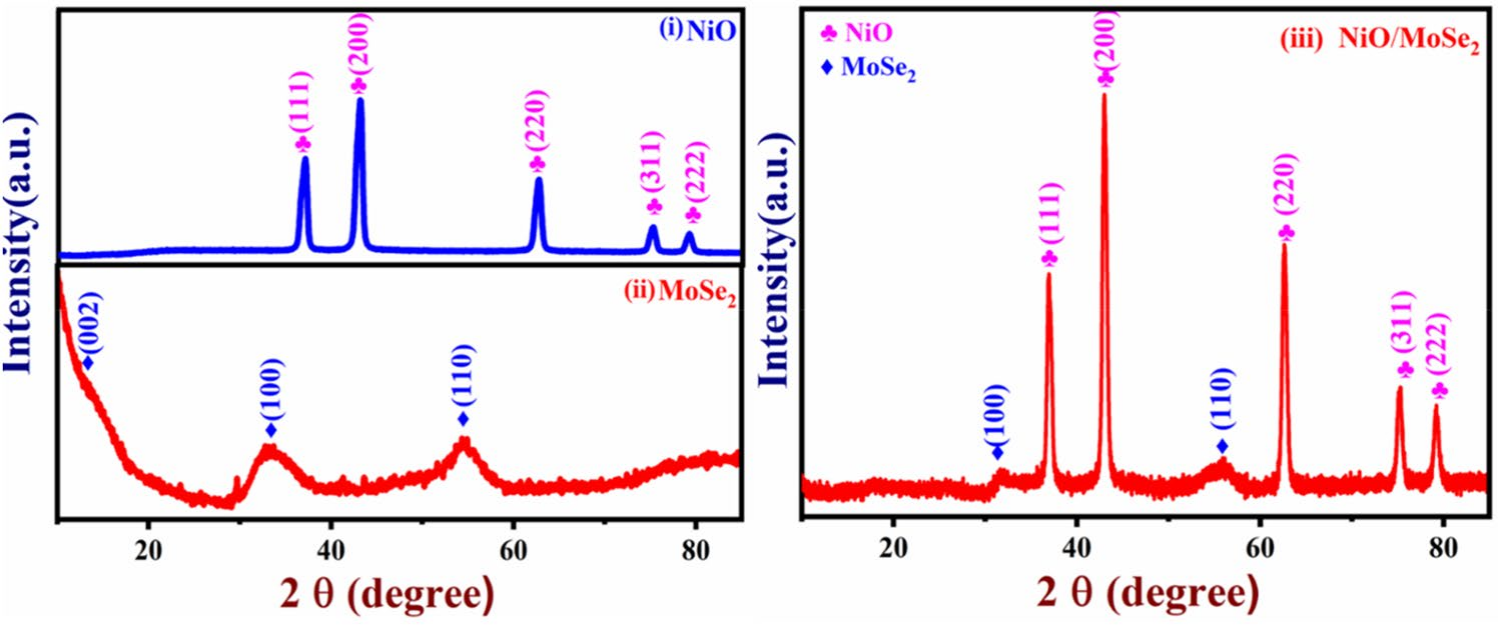
PXRD spectra of the (i) NiO, (ii) MoSe_2_ powder and (iii) NiO/MoSe_2_ nanocomposite.

The crystallinity of MoSe_2,_ NiO, and NiO/MoSe_2_ nanocomposite were studied by using PXRD. For the NiO sample, XRD bands were observed at 37.05°, 43.07°, 62.64°, 75.19° and 79.15° which were assigned to (111), (200), (220), (311) and (222) planes of NiO (Fig. 1, curve i). PXRD spectrum of MoSe_2_ exhibited diffraction peaks at 13.44°, 33.70° and 54.34° which were related to the (002), (100) and (110) planes, respectively (Fig. 1, curve ii). This XRD data confirmed that the hexagonal 2H-MoSe_2_ phase formation was obtained (JCPDS No. 29-0914)^42^. The XRD spectrum of NiO/MoSe_2_ nanocomposite was showed two major diffraction peaks at 32.44° and 54.98° which were corresponded to the MoSe_2_ planes of (100) and (110), respectively. In addition, NiO diffraction peaks were also observed at 36.95°, 43.07°, 62.61°, 75.25° and 79.32° due to the crystal planes of (111), (200), (220), (311) and (222) which confirmed that crystalline cubic NiO nanorods were synthesized (JCPDS No. 71-1179)^43^ (Fig. 1, curve iii). PXRD spectra of MoSe_2_ did not show very sharp XRD peaks which may be due to the presence of semi-crystalline MoSe_2_ in the composite. Furthermore, the PXRD results indicated that NiO/MoSe_2_ nanocomposite was successfully synthesized by hydrothermal method.

### Surface topography analysis of NiO/MoSe_2_ composite

**Figure 2:**
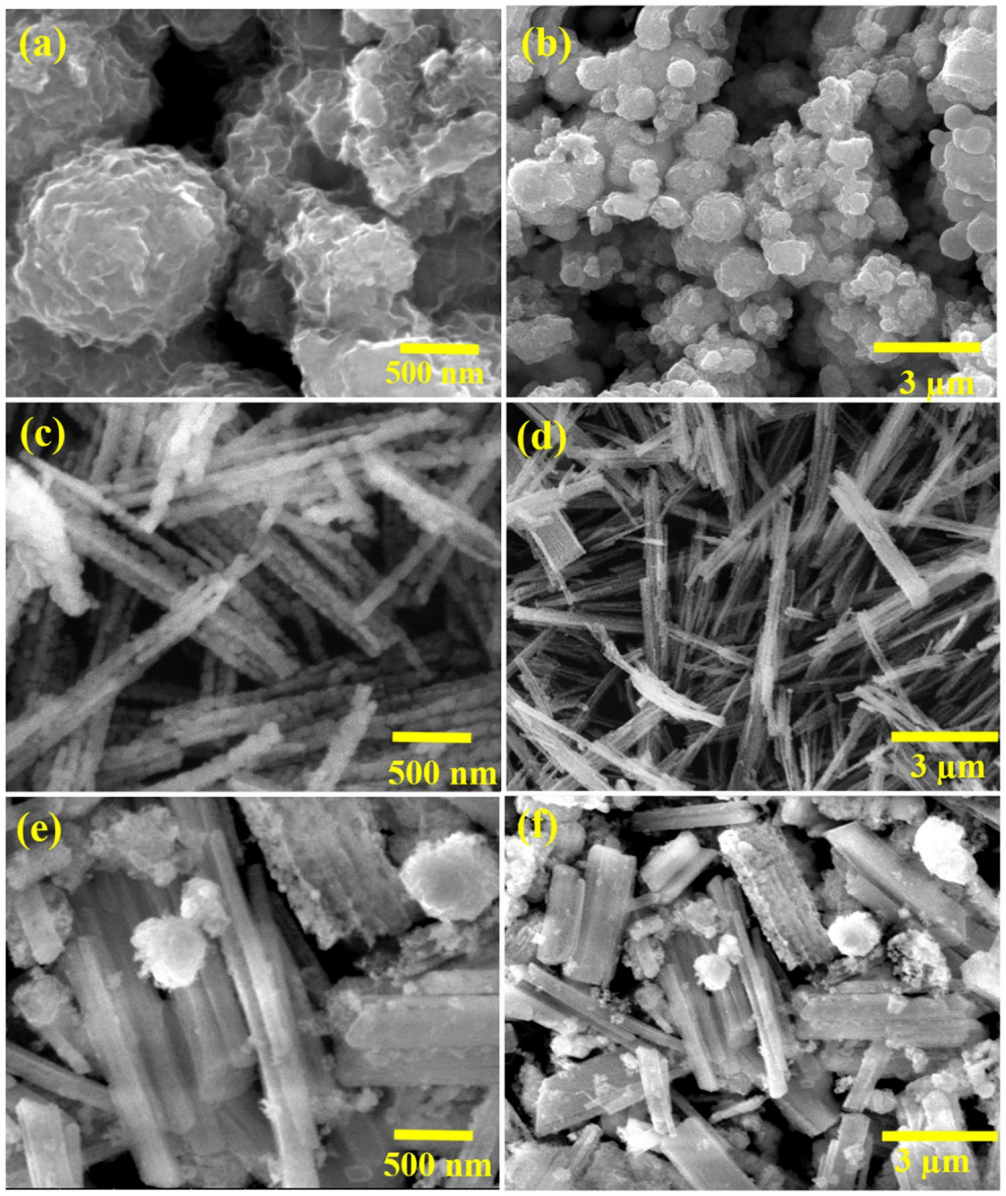
FE-SEM images of (**a**,**b**) MoSe_2_, (**c**,**d**) NiO nanorods and (**e**,**f**) NiO/MoSe_2_ nanocomposite.

The topography features of MoSe_2_, NiO and NiO/MoSe_2_ nanocomposite have been investigated by FE-SEM at different magnifications (Fig. 2a–f). As-prepared MoSe_2_ exhibited nanoflowers-like structure (Fig. 2a,b)^42^. FE-SEM images of NiO were recorded as shown in Fig. 2c,d, which confirmed the presence of large number of nanorods with the average lengths of 4 to 8 µm and the average diameter of nanorods was ~ 15 nm. It was noted that NiO nanorods were formed uniformly with high surface area (Fig. 2c,d). In addition, from the surface morphology of the NiO/MoSe_2_ nanocomposite, it was confirmed that MoSe_2_ layers were incorporated with NiO nanorods (Fig. 2e,f).

**Figure 3:**
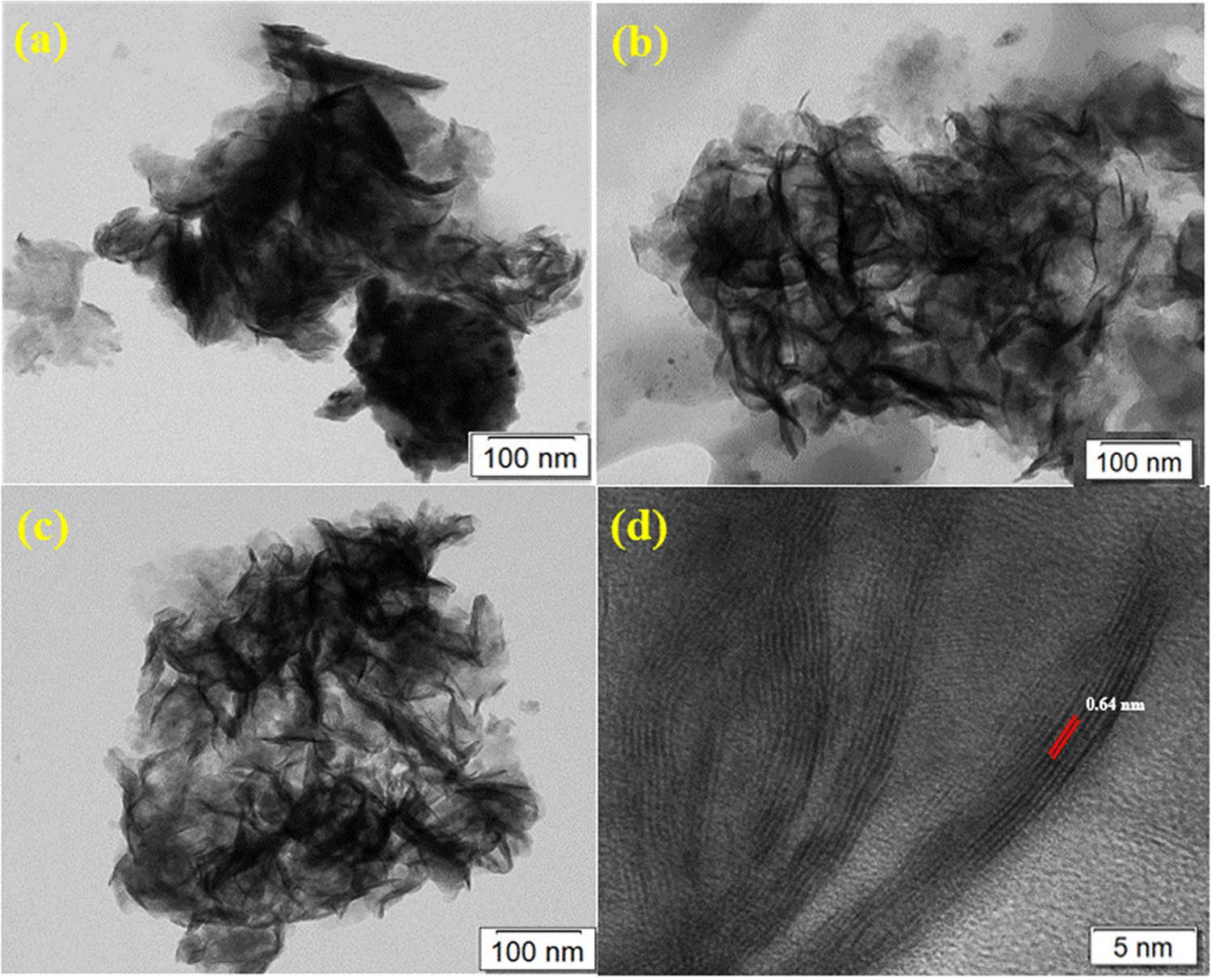
(**a**–**c**) HR-TEM images of MoSe_2_ nanosheets and (**d**) the lattice fringe corresponding to the *d* spacing of MoSe_2_ nanosheets.

**Figure 4:**
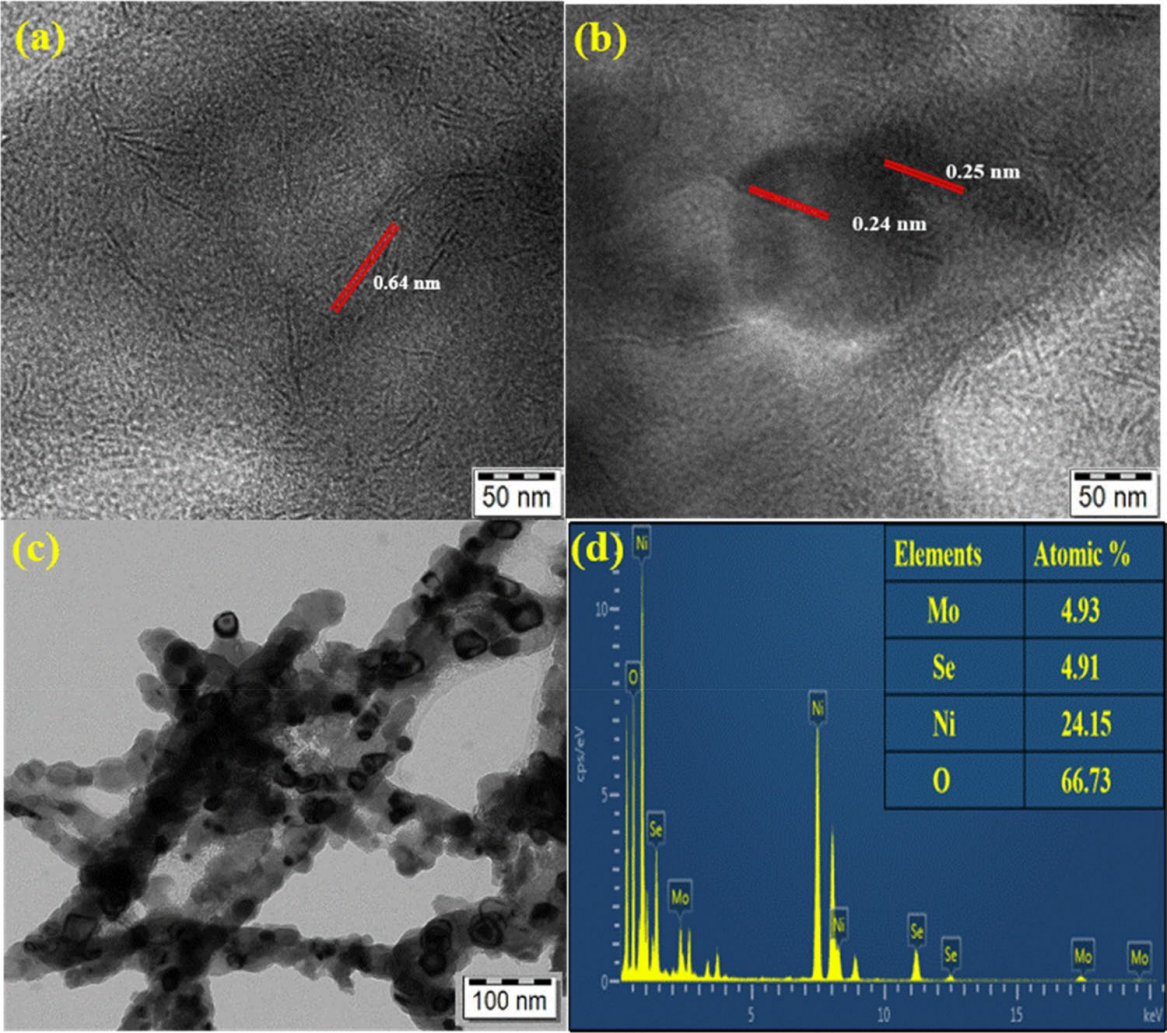
(**a**,**b**) High-resolution HR-TEM images of NiO/MoSe_2_ with the measured lattice spaces. (**c**) HR-TEM image and (**d**) EDX spectrum of the NiO/MoSe_2_.

HR-TEM could provide more information about the elemental and compound structures on the atomic scale of MoSe_2_ and NiO/MoSe_2_ composite. As shown in Fig. 3a–d, agglomerated MoSe_2_ nanosheets were observed in the bulk sample. The lattice fringes of MoSe_2_ nanosheets were measured as 0.64 nm which was in good agreement with the (002) plane (Figs. 3d and 4a)^44^.

In addition, the lattice fringes of NiO were measured as 0.24 and 0.25 nm by high-resolution TEM image analysis^45^ (Fig. 4b). HR-TEM images of NiO/MoSe_2_ nanocomposite had indicated that MoSe_2_ nanosheets were incorporated with NiO nanorods (Fig. 4c). Next, EDX analysis was carried out on NiO/MoSe_2_ nanocomposite which revealed the chemical composition [4.93% Mo, 4.91% Se, 24.15% Ni and 66.73% O] of the materials that successfully confirmed the formation of NiO/MoSe_2_ nanocomposite (Fig. 4d).

### X-ray photoelectron spectroscopy (XPS) analysis of NiO/MoSe_2_

**Figure 5:**
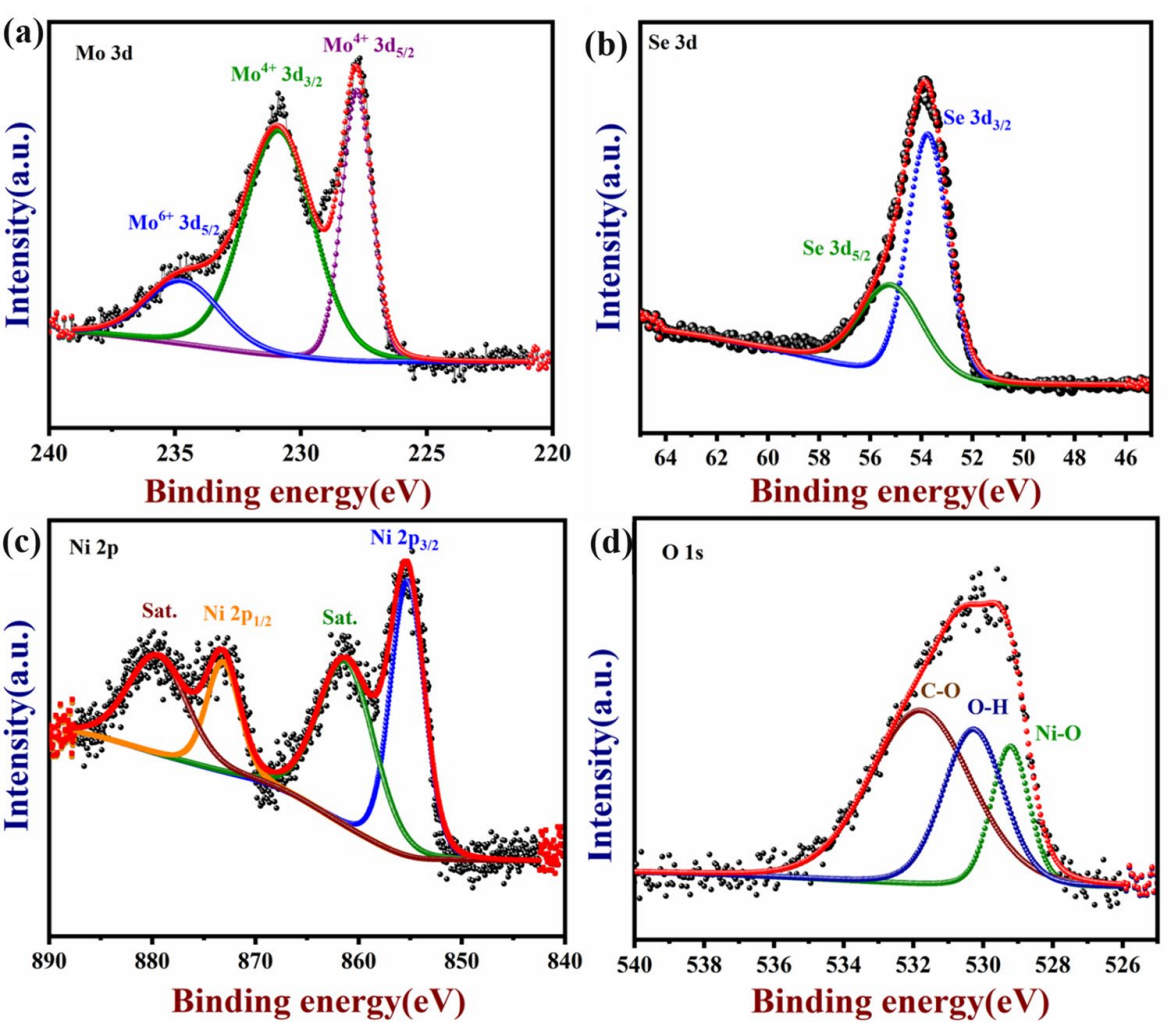
XPS spectra of the NiO/MoSe_2_ nanocomposite were recorded: (**a**) Mo 3*d*, (**b**) Se 3*d*, (**c**) Ni 2*p* and (**d**) O 1* s*.

XPS was widely used to investigate the elemental composition and understand the exact details about the electronic state of the elements^46^. The wide scan XPS spectrum was recorded for NiO/MoSe_2_ sample which showed the major peaks of Ni, O, Mo, and Se elements in their respective electronic states (Fig. S1a, b). From the full-wide (0–1000 eV) scanned survey spectrum (Fig. 5a–d), Ni, O, Mo, and Se present in the NiO/MoSe_2_ nanocomposite were confirmed and indicated the high-purity of the synthesized nanocomposite. Moreover, XPS spectra of Mo 3d was deconvoluted into three major peaks 227.8, 231.1, 253.eV corresponding to the various electronic states of Mo^4+^ 3*d*_5/2_, Mo^4+^ 3*d*_3/2_ and Mo^6+^ 3*d*_5/2_, respectively^21^ (Fig. 5a). Figure 5b showed two peaks with binding energies of 53.8 and 55.3 eV corresponding to the divalent Se ions (Se 3*d*_5/2_ and 3*d*_3/2_, respectively). These XPS results were consistent with the earlier reports on the valence states of the MoSe_2_^20^_._ In addition, Ni 2*p* spectrum was displayed two edge splits by spin–orbital coupling of the 2*p*_3/2_ main peak at 854.7 eV (Fig. 5c) and its satellite peak at 861.9 eV. The 2p_1/2_ main peak of Ni 2*p* at 872.4 eV and its satellite peak at 879.7 eV were proved the existence of NiO^46^. As shown in Fig. 5d, three peaks for the O 1*s* were observed for the binding energies of O–Ni, Ni–O–H and O–C at 529.2 eV, 530.2 eV and 532.1 eV, respectively^47^.

### Electrochemical impedance spectroscopy analysis (EIS)

**Figure 6:**
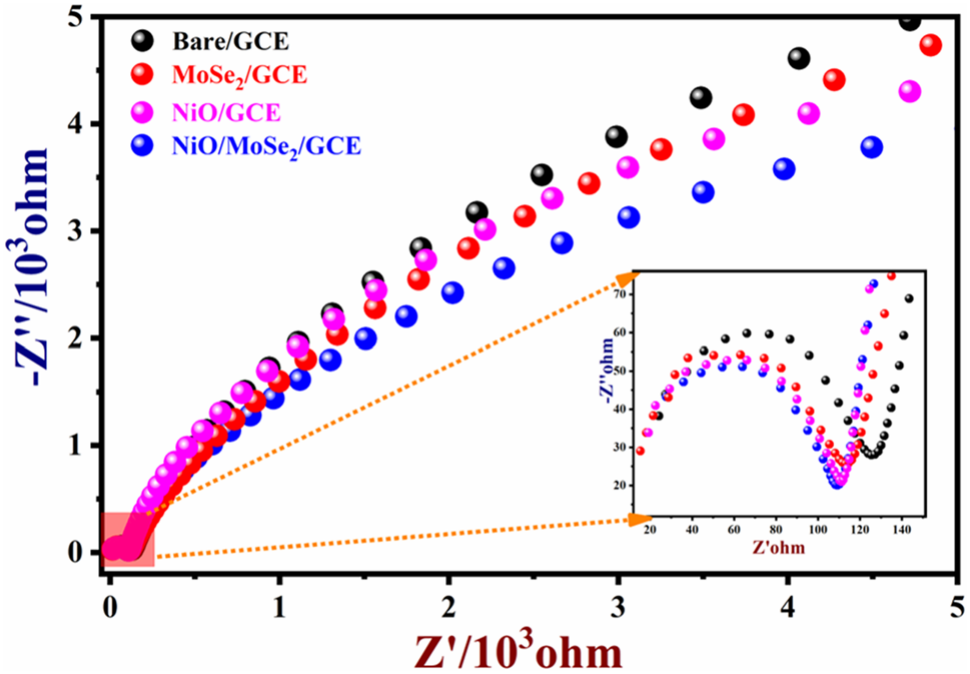
Typical Nyquist plots of NiO/MoSe_2_/GCE, NiO/GCE, MoSe_2_/GCE and bare/GCE were recorded in 0.1 M KCl containing 5 mM [Fe(CN)_6_]^3−/4−^ in the frequency range from 0.1 Hz to 1000 kHz (inset is the enlarged view of high frequency range).

Next, EIS was used to study the charge transfer resistance of the modified electrodes. The Nyquist plots were recorded in 0.1 M KCl containing 5 mM [Fe(CN)_6_]^3−/4−^ using bare GCE, NiO/GCE, MoSe_2_/GCE, and NiO/MoSe_2_/GCE. The charge transfer resistance (R_ct_) of the modified electrode can be estimated at the low-frequency region of the semi-circle from the Nyquist plots. Each of the EIS spectrum is consisted of a typical semicircle and the high-frequency region in the EIS spectrum provided the parametric information about the resistance of the electrode/electrolyte interface^48^. The solution resistance (R_s_) was found to be  13 Ω. After the subtraction of R_s_, the R_ct_ values of the NiO/MoSe_2_/GCE (79.6 Ω), MoSe_2_/GCE (99.4 Ω), NiO/GCE (92 Ω), and bare/GCE (103.4 Ω) were calculated (Fig. 6). It showed that NiO/MoSe_2_/GCE had exhibited lower R_ct_ value due to the enhanced conductivity of the nanocomposite.

### Electro-catalytic oxidation of glucose at NiO/MoSe_2_/GCE

**Figure 7:**
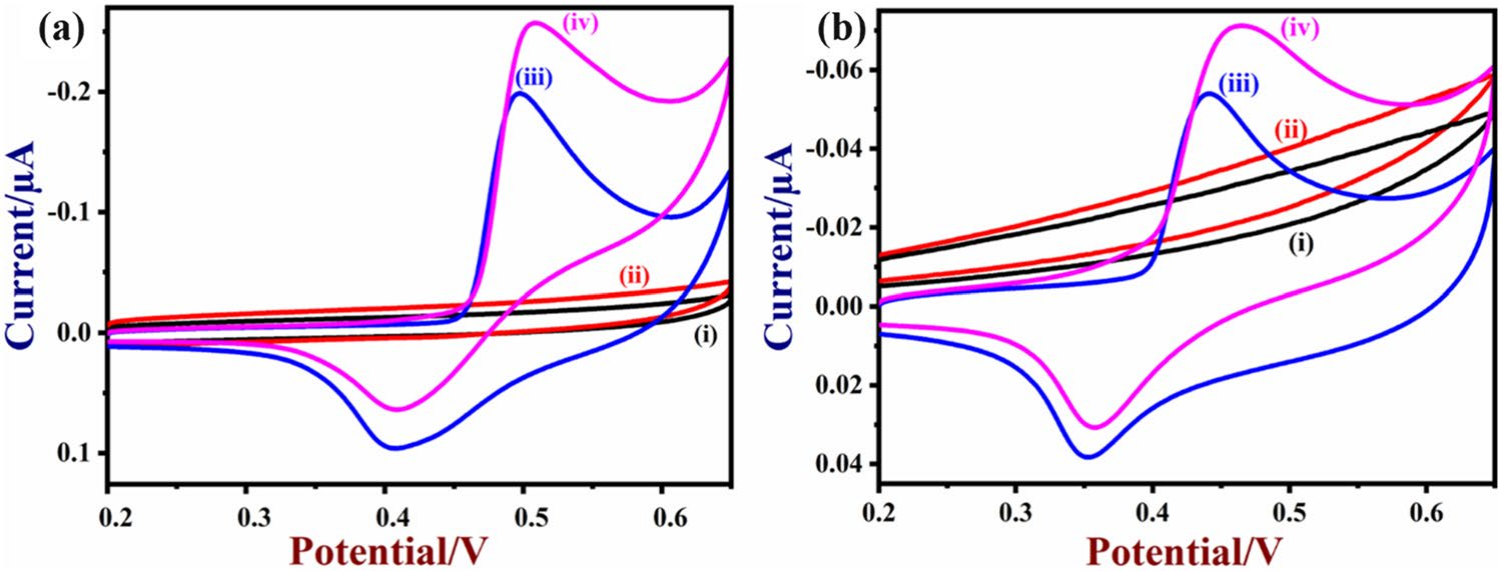
(**a**) CVs of bare-GCE and NiO/MoSe_2_/GCE in 0.1 M NaOH in the absence (curves i and iii) and presence of 50 µM glucose (curves ii and iv). (**b**) CVs of MoSe_2_/GCE and NiO/GCE in 0.1 M NaOH in the absence (curves i and iii) and presence of 50 µM glucose (curves ii and iv). Scan rate = 50 mVs^−1^.

The electro-catalytic activity of the nanocomposite for the glucose oxidation was studied by cyclic voltammetry. Cyclic voltammograms (CVs) of the bare-GCE, NiO, MoSe_2_ and NiO/MoSe_2_ modified GCE’s were recorded in the presence and absence of glucose (50 µM) in 0.1 M NaOH. In the presence of glucose, no oxidation or reduction peak was observed on bare GCE (Fig. 7a, curves i, ii). Interestingly, NiO/MoSe_2_/GCE was showed an enhanced redox peak of Ni^2+^/Ni^3+^ in the potential window between 0.2 and 0.65 V in 0.1 M NaOH. The oxidation and reduction peaks of NiO were appeared at 0.49 V and 0.39 V, respectively (Fig. 7a, curve iii). The formal potential (E°′ = E_pa_ + E_pc_/2) of the NiO redox peak on MoSe_2_ was found to be + 0.44 V which was in agreement with other reported sensors^35^. The peak-to-peak (ΔE_p_ = E_pa _− E_pc_) separation of redox peak (NiO) was found to be 100 mV. It was clear that NiO nanorods were firmly attached with MoSe_2_. Furthermore, it is worth to mention that after the injection of 50 µM glucose in to 0.1 M NaOH, NiO/MoSe_2_/GCE was exhibited a notable enhancement in the anodic peak current at 0.50 V for glucose oxidation and the decrease in the cathodic peak current at ~ 0.40 V. This indicated the good electro-catalytic activity of the NiO/MoSe_2_ modified electrode (Fig. 7a curves iii and iv).

**Scheme 1:**
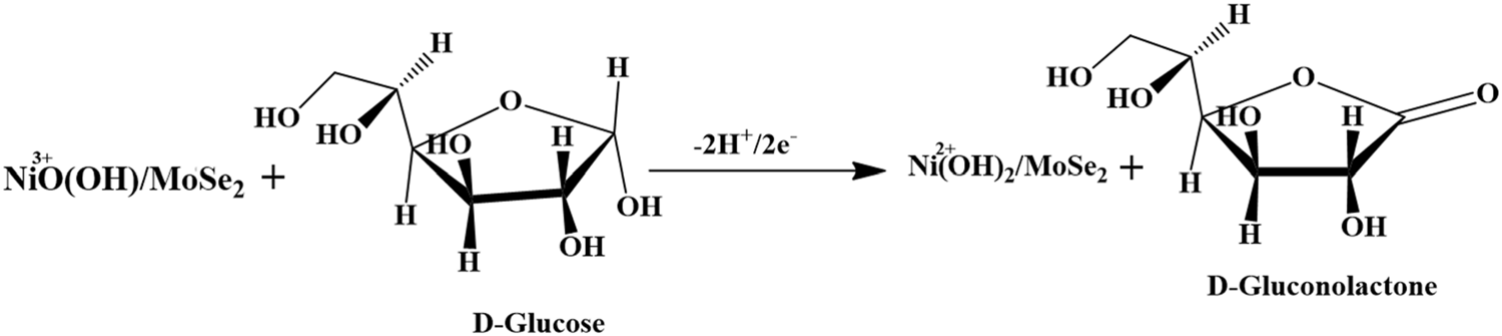
Electro-catalytic oxidation mechanism for glucose on NiO/MoSe_2_ nanocomposite modified electrode.

The observed improvement in the electrocatalytic activity of the NiO/MoSe_2_ nanocomposite for glucose oxidation was further revealed by comparison studies performed with the individually prepared MoSe_2_/GCE and NiO/GCE under the same condition. MoSe_2_/GCE did not show any oxidation peak for glucose (Fig. 7b curves i and ii). However, NiO/GCE was showed an oxidation peak for glucose at 0.46 V (Fig. 7b, curves iii and iv). But, the observed catalytic current was very low compared to the NiO/MoSe_2_ modified GCE (Fig. 7a curve iii and iv). The higher electro-catalytic activity of the nanocomposite might come from the synergistic interaction between NiO and MoSe_2_ (Scheme 1). The proposed mechanism for the electro-catalytic oxidation of glucose on NiO/MoSe_2_/GCE is shown in Eqs. (1, 2)^49^.

1 $${\text{NiO}} + ~{\text{OH}}^{ - } \to {\text{NiO}}\left( {{\text{OH}}} \right) + {\text{e}}^{ - }$$

2 $${\text{NiO}}\left( {{\text{OH}}} \right) + glu\cos e \to {\text{Ni}}\left( {{\text{OH}}} \right)_{2} + gluconolactone$$

During the electrochemical oxidation process, NiO was reacted with the hydroxyl (OH^-^) ions in the alkaline solution (0.1 M NaOH) which converted Ni^2+^ to Ni^3+^. This confirmed the formation of nickel oxyhydroxide (NiOOH) (oxidising agent) and converted glucose in to gluconolactone^49^.

**Figure 8:**
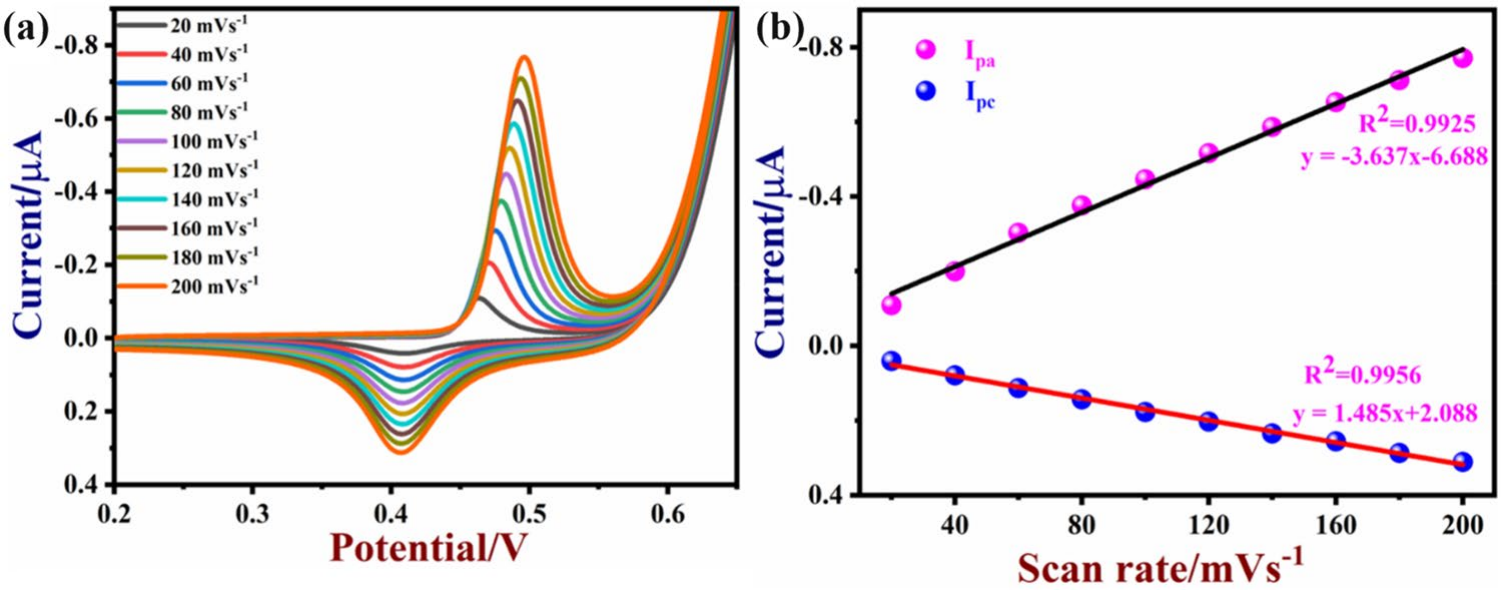
CVs recorded using (**a**) NiO/MoSe_2_/GCE in 0.1 M NaOH containing 50 µM glucose at various scan rates (from 20 to 200 mVs^−1^). (**b**) The linear relationship between the scan rate versus peak currents with a correlation coefficient values of (I_pa,_ R^2^ = 0.9925 and I_pc,_ R^2^ = 0.9956).

The effects of scan rate on the glucose oxidation was also studied by CV. CVs were recorded in 0.1 M NaOH containing 50 µM glucose at different scan rates (from 20 to 200 mVs^−1^) using a NiO/MoSe_2_ nanocomposite modified GCE (Fig. 8a). As can be seen, both cathodic and anodic peaks (I_pc_ and I_pa_) currents were linearly increased with the scan rates. This indicated that glucose oxidation was a surface-controlled process on NiO/MoSe_2_/GCE^50^. A linear relationship was observed between the scan rate (mVs^-1^) and peak currents (I/µA) with a correlation coefficient of (I_pa;_ R^2^ of 0.9925) and (I_pc;_ R^2^ of 0.9956) (Fig. 8b).

**Figure 9:**
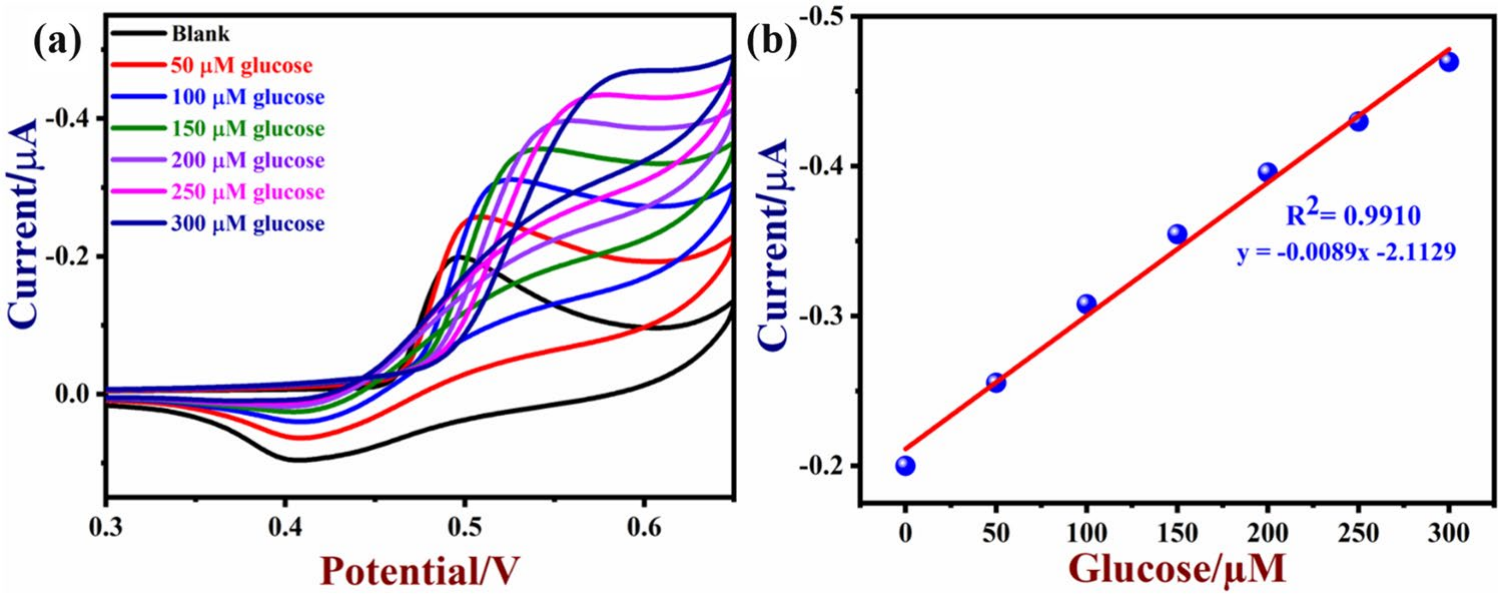
(**a**) CVs of NiO/MoSe_2_/GCE were recorded in the presence of different concentrations of glucose from 50 to 300 µM in 0.1 M NaOH at a scan rate 50 mVs^−1^. (**b**) The corresponding calibration graph of glucose with different concentrations.

Next, CVs were recorded in 0.1 M NaOH with different concentrations of glucose (from 50 to 350 µM) using a NiO/MoSe_2_/GCE (Fig. 9a, b). The oxidation peak currents of NiO/MoSe_2_/GCE were increased linearly with the concentrations of the glucose. In addition, the glucose oxidation peaks were slightly shifted to positive potential because of the restricted diffusion-controlled and mass transfer process^51^. It may be also due to the local pH change in the electrolyte during the oxidation of glucose and the formation of some oxidized intermediates.

Next, the optimum amount of NiO/MoSe_2_ catalyst on the GCE and its effect on the electrocatalytic oxidation of 50 µM glucose was studied (Fig. S2a). For this purpose, different volumes (10 to 50 µL) of NiO/MoSe_2_ dispersion (0.2 mg/mL) was drop-casted on GCE (Fig. S2b). It was found that glucose oxidation was kinetically favourable on NiO/MoSe_2_/GCE coated with 2 μg (10 µL) of catalyst. However, higher loadings of the NiO/MoSe_2_ material (4, 6, 8, and 10 μg) on the GCE were negatively affected the glucose oxidation current (decreased). It might be due to the higher amount of the catalyst was not favourable for the interaction between glucose and electrode surface. From this study, 2 μg (10 µL) of NiO/MoSe_2_ was selected to prepare modified GCE for further studies (Fig. S2a, b).

### Amperometric detection of glucose

Amperometry is one of the highly sensitive electrochemical techniques which works at constant applied potential and current responses were recorded with time by varying the concentrations of the analyte. Firstly, the optimum voltage for glucose oxidation was determined from the series of amperograms recorded with the addition of glucose from 50 to 300 µM at varied applied voltages (from 0.4, 0.45, 0.50 and 0.55 V) on NiO/MoSe_2_/GCE (Fig. S3). It was found that NiO/MoSe_2_/GCE was well responded with high current for the each additions of glucose at 0.5 V (Fig. S3, red curve), so it was selected as the optimum voltage for further investigations (Fig. S3).

**Figure 10:**
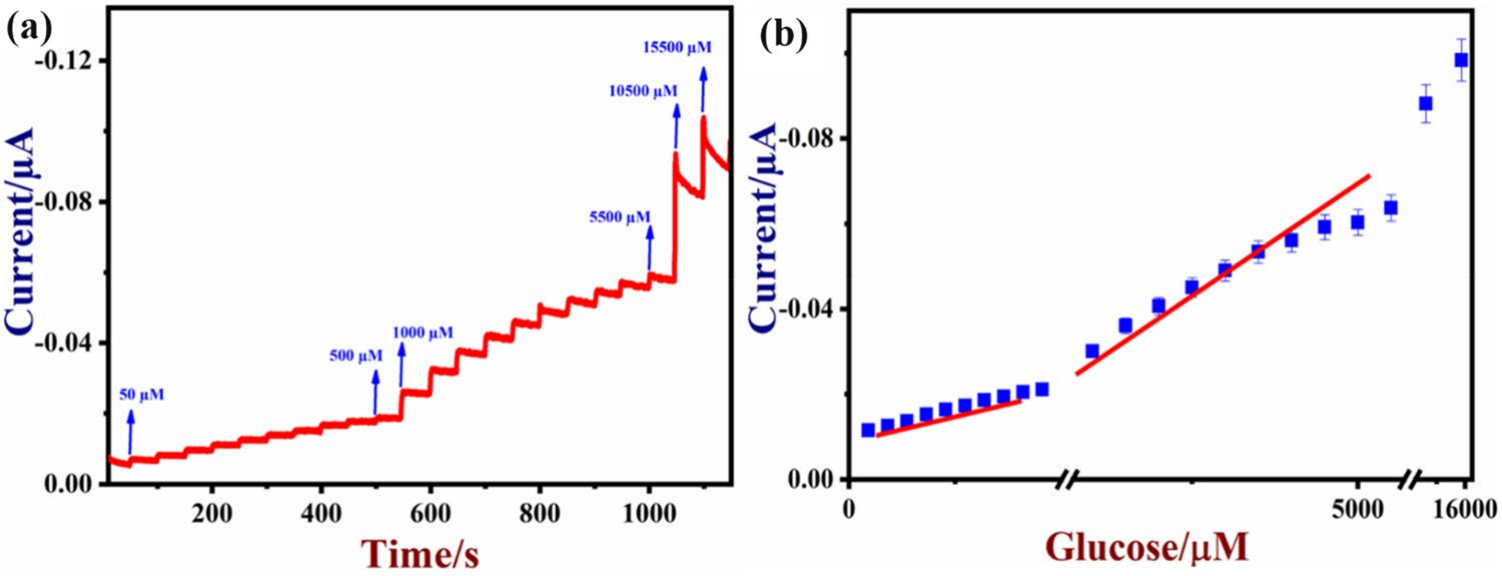
(**a**) Amperometric curve was recorded using a NiO/MoSe_2_/GCE with the successive additions of glucose from 50 µM to 15.5 mM at 0.5 V in 0.1 M NaOH. This solution was stirred at 1200 rpm. (**b**) The corresponding calibration plot of glucose with the standard deviation (n = 3).

Figure 10a shows the amperograms recorded in 0.1 M NaOH with different concentrations of glucose. After the injection of different concentrations of glucose (from 50 µM to 15.5 mM), NiO/MoSe_2_/GCE was linearly responded and the steady state current was reached within 2 s after the each addition (Fig. 10a). From this amperograms, a calibration graph was plotted for glucose after triplicate measurements (Fig. 10b) and the corresponding error bars were provided on the calibration plot. As can be seen, a linear relationship was observed between I_pa_ and glucose concentrations with a correlation coefficient of (R^2^) 0.9842 (Fig. 10b).

Generally, the limit of detection (LOD) was calculated from formula of 3× standard deviation of the response/slope of the calibration graph. The standard deviation (SD) of the blank was 2.46 × 10^–7^ A and the slope of the calibration curve was 1.25 × 10^–6^ A µM^−1^. Using these values, LOD was estimated as 0.6 µM (S/N = 3). From the above results, it was concluded that NiO/MoSe_2_/GCE had exhibited a wide linear range of detection and lower LOD due to the high electron transfer rate between glucose and NiO/MoSe_2_/GCE in 0.1 M NaOH. In addition, NiO/MoSe_2_/GCE sensor was also showed more promising analytical performance for glucose sensing compared to some of the reported electrochemical sensors (Table 1).

**Table 1:** Comparison of the analytical performance of the NiO/MoSe_2_ electrode with the other reported glucose sensors based on different electrode materials.

Electrode	Electrolyte	Applied potential (V)	Liner range of glucose (mM)	LOD (µM)	References
Cu_2_O/CuE	0.1 M NaOH	0.6	0.05 to 6.75	37	^52^
Pt–Au/Au–Si	0.1 M PBS	0.3	0 to 1.05	6.0	^53^
CuO/GE	0.1 M NaOH	0.6	0.004 to 8	4.0	^54^
Nanoporous Pt thin film	PBS	0.4	1 to 10	97	^55^
Cu NB/CuE	50 mM NaOH	0.6	–	10	^56^
Ni/NiO-Nafion-rGO/SPE	0.1 M NaOH	0.55	0.03 to 6.44	1.8	^39^
NiO/GNS/GCE	0.1 M NaOH	0.5	0.005 to 4.2	5.0	^37^
NiO/MWCNTs/Ta	0.1 M NaOH	0.5	0.001 to 7	2.0	^38^
NiO/MoS_2_/GCE	0.1 M NaOH	0.55	0.01 to10	1.6	^57^
NiO/MoSe_2_/GCE	0.1 M NaOH	0.5	0.05 to 15.5	0.6	This work

*NB* nanobelt, *NiO/GNS* nickel oxide/graphene nanosheet, *MWCNTs* multi-walled carbon nanotubes, *CuE* Copper electrode, *Au–Si* gold coated silicon substrate, *GE* graphite electrode, *GCE* glassy carbon electrode, *SPE* screen printed electrode, *PBS* phosphate buffer solution, *Ta* Tantalum.

### Interference, repeatability and stability analysis

**Figure 11:**
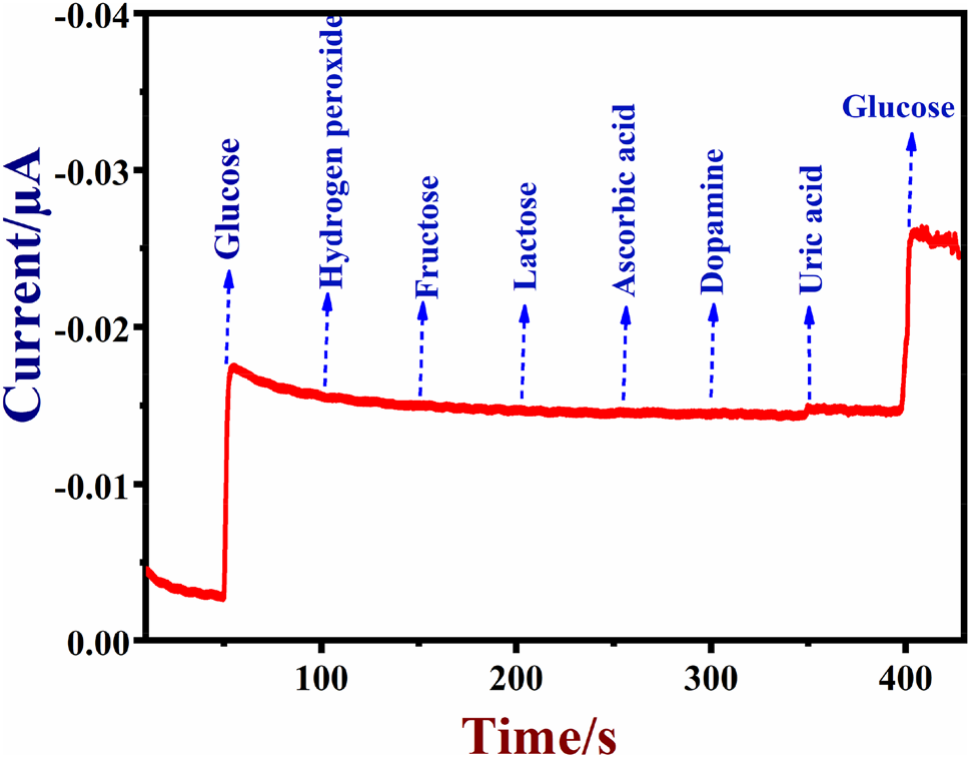
Amperometric response were recorded using a NiO/MoSe_2_/GCE in 0.1 M NaOH in the presence of H_2_O_2_ (0.1 mM), fructose (0.1 mM), lactose (0.1 mM), ascorbic acid (0.1 mM), uric acid (0.1 mM), dopamine (0.1 mM) and glucose (0.5 mM). Rotation rate was 1200 rpm. The applied potential was 0.5 V.

The selectivity of the NiO/MoSe_2_/GCE was tested in the presence of other common biological compounds because they could affect the sensor response in the real samples. In order to use NiO/MoSe_2_/GCE sensor in real-world samples, NiO/MoSe_2_/GCE was tested with the important biomolecules (H_2_O_2_, fructose, lactose, DA, AA, and UA) which may affect the direct electrochemical oxidation of glucose because of their overlapping oxidation potentials with glucose. As shown in Fig. 11, after the each addition of interferent compounds (each 0.1 mM) such as H_2_O_2_, fructose, lactose, UA, DA, and AA, the NiO/MoSe_2_/GCE did not show any observable current response at 0.5 V (Fig. 11). It indicated the good selectivity of the modified electrode.

Moreover, repeatability and stability of NiO/MoSe_2_/GCE were also investigated by cyclic voltammetry. CVs were recorded in 0.1 M NaOH containing 50 µM glucose for five times in the interval of 0 to 8 h (Fig. S4a, b). The relative standard deviation (RSD) for five repeated measurements was 2.08%. This data showed that NiO/MoSe_2_/GCE can be used for continuous glucose measurements.

The stability of the NiO/MoSe_2_ film on the GCE surface was also tested by recording continuous CVs in 0.1 M NaOH for 50 cycles (Fig. S5a). The anodic and cathodic peak currents of NiO/MoSe_2_/GCE were slightly decreased about 9%. However, the redox potential of the NiO/MoSe_2_/GCE did not change significantly after scanning about 50 cycles that confirmed the good stability of the modified electrode (Fig. S5a, b). Next, the reproducibility of NiO/MoSe_2_/GCE was investigated by detecting 50 μM glucose under the same condition using three independently prepared NiO/MoSe_2_/GCE electrodes. The relative standard deviation (RSD) for the three different electrodes was 5.3% which showed that the electrode modification procedure was highly reproducible (Fig. S6).

### Determination of glucose in blood serum samples

The real application of the NiO/MoSe_2_/GCE was tested by detecting glucose concentrations in blood serum samples. The human blood serum samples were obtained from the SRM Medical College Hospital and Research Centre which is located inside our campus. The serum was obtained using the following procedure. The blood was collected in a serum separator tube (SST, tiger top tube) from two healthy individuals and allowed to clot for one hour at room temperature. After that, the sample was centrifuged at 2500 rpm for 15 min to remove the clot. Finally, the resulted serum liquid was stored at − 20 °C in the refrigerator^58^. Next, 100 µL of the blood serum solution was injected into the 10 mL of 0.1 M NaOH and the electrode response was recorded at 0.5 V^59^. The blood glucose concentration in the human blood serum was estimated from the calibrated graph (Fig. 9b). Our obtained glucose concentrations in two different serum samples were shown in Table 2. It has been observed that our proposed sensor showed accurate results compared to the results obtained from the SRM Hospital and Research Centre (Table 2). Thus, we have concluded that our proposed sensor may be useful to construct commercial glucose sensing devices.

**Table 2:** Determination of glucose in human blood serum samples by using a NiO/MoSe_2_/GCE.

S. No	Samples	Glucose concentration (mM) detected by hexokinase method (from SRM Hospital)	Glucose concentration (mM) detected by NiO/MoSe_2_/GCE	Recovery %	Error %
1	Human serum-1	7.8	7.6	97.4	2.6
2	Human serum-2	9.4	9.29	98.8	1.2

The long-term stability of the NiO/MoSe/GCE was also tested by using the same modified electrode for the determination glucose in 50 µL blood serum. During this period, CVs were recorded with blood serum in 0.1 M NaOH from day 1 to 25 days. The oxidation current of glucose in blood serum was decreased by about 4.3% (n = 5) after 25 days which indicated that NiO/MoSe/GCE may be useful for repeated measurements (Fig. S7).

## Conclusions

In summary, MoSe_2_ incorporated NiO nanorods were hydrothermally synthesized and comprehensively characterized by PXRD, HR-TEM, FE-SEM and XPS. It was found that MoSe_2_ nanosheets were present on the NiO nanorods. In addition, the electrochemical and electrocatalytic properties of NiO/MoSe_2_ have been studied which showed that this sensor may be useful for selective detection of glucose by amperometry. The NiO/MoSe_2_ catalyst loading (2 µg on GCE) and applied voltage (0.5 V) for glucose oxidation were optimized. The NiO/MoSe_2_/GCE exhibited a linear response for the detection of glucose from 50 μM to 15.5 mM and LOD was 0.6 µM. Furthermore, stability, reproducibility and repeatability studies were indicated that the NiO/MoSe_2_/GCE was highly stable and can be used for repeated measurements. The response time of the sensor was 2 s for glucose. The real sample analysis was also carried out in blood serum samples using the NiO/MoSe_2_/GCE. The glucose recovery analysis were indicated that NiO/MoSe_2_/GCE can be applied for the detection of glucose in real samples with high selectivity and accuracy. Based on our results, NiO/MoSe_2_ nanocomposite-based electrode can be easily prepared for the selective detection of glucose in various samples.

## Experimental

### Reagents and apparatus

All the reagents and chemicals used were of analytical grade. Ammonium molybdate tetrahydrate (NH_4_)_6_ Mo_7_O_24_.4H_2_O, selenium metal powder (99.9%), hydrazine hydrate (N_2_H_4_, 80%), nickel chloride hexahydrate (NiCl_2_.6H_2_O), sodium hydroxide (NaOH), sodium oxalate (Na_2_C_2_O_4_) and glucose (C_6_H_12_O_6_)) were purchased from Sigma-Aldrich, Sisco Research Laboratories, Thermo Fisher Scientific and Loba Chemie. These chemicals were used without any further purifications. All solutions were prepared with Mili-Q distilled water (18.2 MΩ cm @ 25 ± 2 °C).

Electrochemical measurements were carried out by using the electrochemical workstation (Model: CHI-760E) from CH Instruments, Austin, TX, USA. Electrochemical studies were performed in a standard electrochemical cell using a three-electrode system with NiO/MoSe_2_/GCE as the working electrode, Ag/AgCl (3M KCl) as the reference electrode and platinum wire as the counter (auxiliary) electrode. The blood serum samples were received from the SRM Medical College Hospital and Research Centre, Kattankulathur, Tamil Nadu. All the experiments were carried out in accordance with the relevant guidelines and regulations. The SRMIST ethics committee was approved the experiments (Ref. No: 002/HYC/IEC/2018). Informed consents were obtained from the human participants of this study.

### Hydrothermal synthesis of MoSe_2_ nanosheets

MoSe_2_ nanosheets were prepared by the hydrothermal method as reported elsewhere with some modifications^12^. Briefly, 0.03 g of (NH_4_)_**6**_Mo_**7**_O_24_·4H_2_O was added in to 50 mL of distilled water and stirred for 20 min. 0.07 g of selenium (Se) metal powder was dissolved in 10 mL distilled water and 2 mL of 80% hydrazine hydrate solution was added. This solution was mixed well with constant stirring (1000 rpm) up to 24 h. After that, the Se solution was slowly added into (NH_4_)_2_.MoO_4_ solution which produced an orange-red colour. Later, the solution mixture was transferred into the Teflon-lined autoclave and heated in a hot air oven at 200 °C for 12 h. Finally, the sample was centrifuged at 5000 rpm for 15 min and the precipitate was collected, washed with ethanol, water and dried in hot air oven at 60 °C. The obtained product was annealed in a tubular furnace at 500 °C for 3 h under nitrogen atmosphere and a black MoSe_2_ powder was obtained.

### Synthesis of NiO/MoSe_2_ nanocomposite

**Scheme 2:**
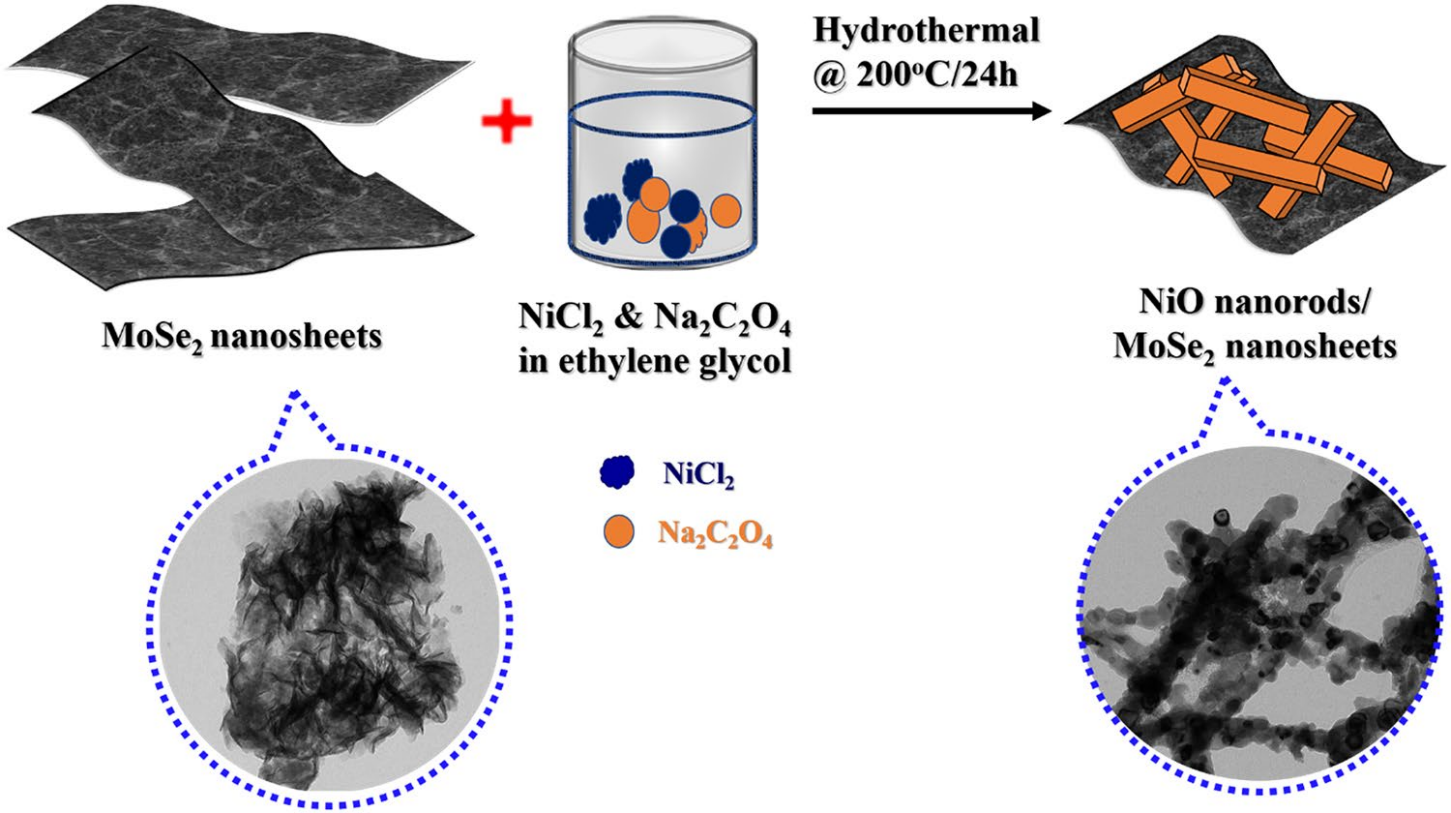
The schematic illustration for the preparation of NiO/MoSe_2_ nanocomposite by hydrothermal method.

NiCl_2_ (0.474 g) and MoSe_2_ (0.307 g) were dissolved in 18 mL of distilled water plus 30 mL ethylene glycol (80%) and stirred continuously for 30 min. After that, Na_2_C_2_O_4_ (0.1206 g) was added in to the above solution under constant stirring at room temperature. Finally, the resulted solution mixture was transferred into a Teflon–lined stainless steel autoclave and maintained at 200 °C for 24 h^60^. Then, the reaction mixture was cooled down to the room temperature and transferred in to a centrifuge tube. After the centrifugation at 8000 rpm for 15 min, the precipitate was collected and dried at 60 ºC for 12 h. After that NiO/MoSe_2_ nanocomposite was calcinated at 500º C for 3 h under N_2_ atmosphere (Scheme 2).

### Material characterizations

The crystal structure of the NiO/MoSe_2_ was investigated by using a powder X-ray diffraction (PXRD) spectrometer with Cu Kα radiation (λ = 0.15406 nm) (X’pert powder XRD system, Malvern Panalytical). The surface morphology of the samples were characterized by using FE-SEM (FEI Quanta FEG 200) and HR-TEM (TEM, JEM-2100 Plus, Jeol) with energy dispersive X-ray (EDX) analysis. For TEM characterizations, 3 µL suspension of (0.5 mg/mL) NiO/MoSe_2_ was drop casted on copper grid and dried at room temperature. The sample coated copper grid was used for HR-TEM analysis. A PHI Versa Probe III Scanning XPS Microprobe was used for the XPS analysis (Physical Electronics, USA).

### Preparation of NiO/MoSe_2_ modified GCE

The glassy carbon electrode (GCE, diameter 3 mm) polished on alumina slurry with different particle sizes (0.05, 0.1, 0.3 µm) and washed with distilled water and ethanol to obtain a mirror-like surface. After that, 1 mg of NiO/MoSe_2_ nanocomposite was dispersed in 5 mL of distilled water and bath sonicated for 30 min. To prepare NiO/MoSe_2_/GCE, 10 μL of NiO/MoSe_2_ composite solution was drop casted on the GCE surface and water was evaporated at 50 °C. For comparison measurements, bare GCE, NiO/GCE, and MoSe_2_/GCE were also similarly prepared.

## Supplementary Information

Supplementary Information.
