# Supplementary material for: Fabrication of 2D-MoSe2 incorporated NiO Nanorods modified electrode for selective detection of glucose in serum samples
Source: Sci Rep. 2021 Jun 24;11:13266. doi: 10.1038/s41598-021-92620-2 (PMC8225789; doi:10.1038/s41598-021-92620-2)
Supplement: Supplementary file 1 — Supplementary Information. [file 41598_2021_92620_MOESM1_ESM.docx]

**Supplementary Information**

**Fabrication of 2D-MoSe_2_ Incorporated NiO Nanorods Modified Electrode for Selective Detection of Glucose in Serum Samples**

# Gayathri Jeevanandham^1^, Kumaran Vediappan^1^, Zeid A. ALOthman^2^, Tariq Altalhi^3^, and Ashok K. Sundramoorthy^*,1^

^1^Department of Chemistry, SRM Institute of Science and Technology, Kattankulathur -603203, Tamil Nadu, India

^2^Chemistry Department, P. O. Box 2455, College of Science, King Saud University, Riyadh 11451, Saudi Arabia

^3^Department of Chemistry, College of Science, Taif University, P.O. Box 11099, Taif 21944, Saudi Arabia

*Corresponding author:

Email: [ashokkus@srmist.edu.in](mailto:ashokkus@srmist.edu.in)

# ^Figures^


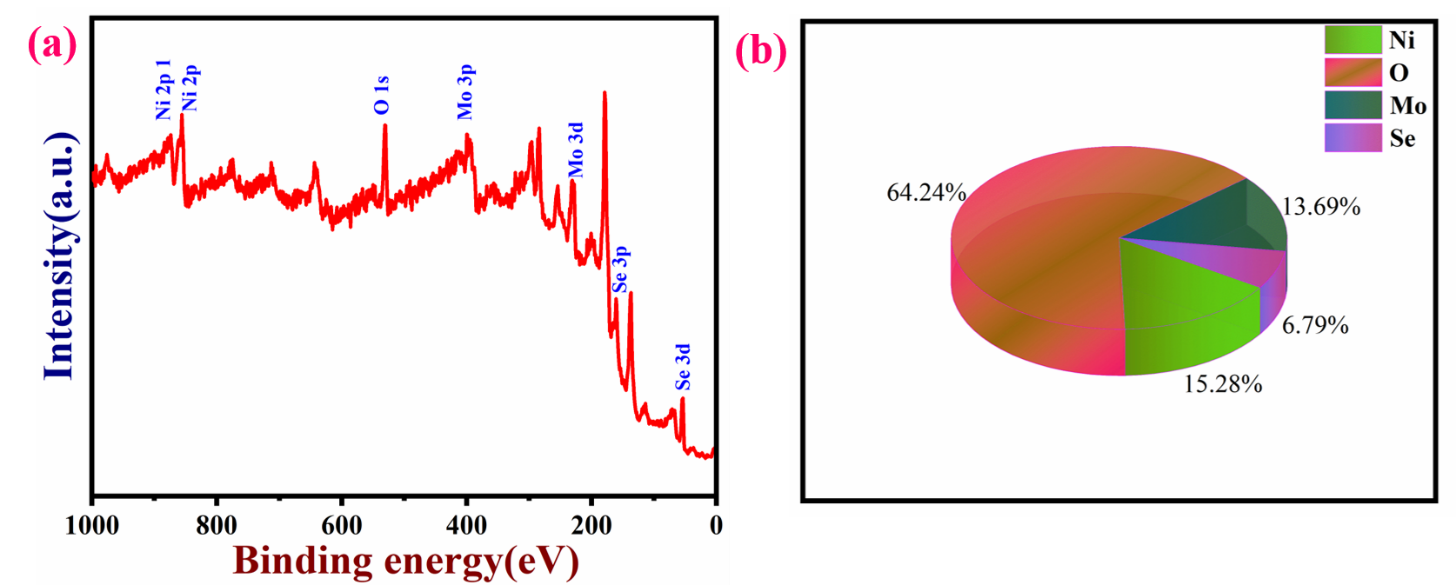


**Fig. S1** XPS spectra of NiO/MoSe_2_. (a) Survey spectrum and (b) pie chart representation of the atomic percentages of elements present in NiO/MoSe_2._


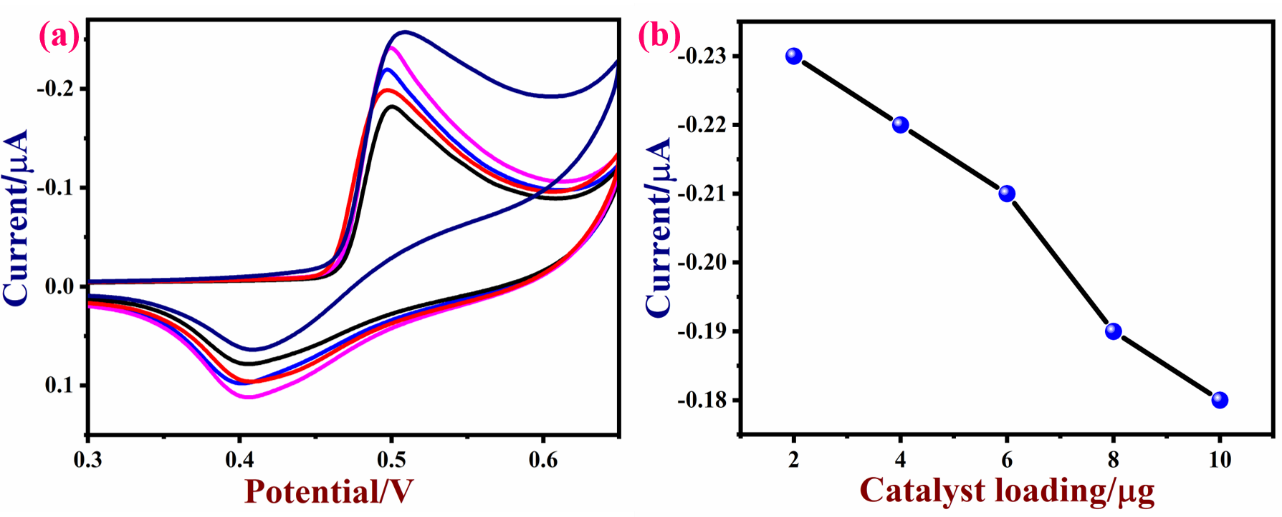


**Fig. S2** (a) CVs were recorded using NiO/MoSe_2_/GCE with different amounts of catalyst loaded from the stock solution (0.2 mg/mL) as 2 (10 μL), 4 (20 μL), 6 (30 μL), 8 (40 μL) and 10 μg (5 μL) for the oxidation of 50 µM glucose. (b) The plots of glucose oxidation currents (I_pa_) *vs.* various amounts of catalyst modified GCE’s.





**Fig. S3** Amperometry curves were recorded using the NiO/MoSe_2_/GCE with successive additions of glucose (50 to 300 µM) at an applied potential of (0.4 to 0.55 V.) in 0.1 M NaOH. The rotation rate = 1200 rpm.


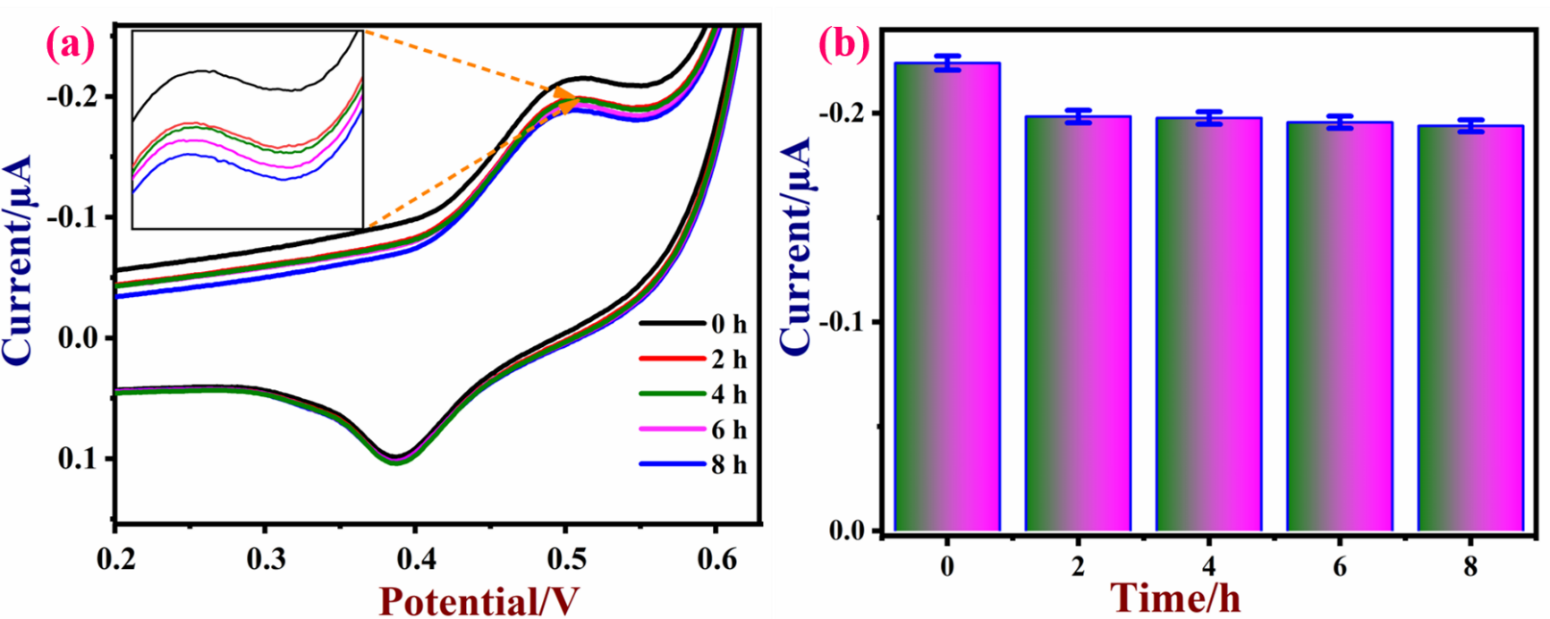


**Fig. S4** (a) CVs of a NiO/MoSe_2_/GCE were recorded in 0.1 M NaOH containing 50 µM of glucose at different time intervals of 0, 2, 4, 6, and 8 h at a scan rate of 50 mVs^-1^. (b) The histogram representation of changes in currents with time and corresponding error bars are displayed.


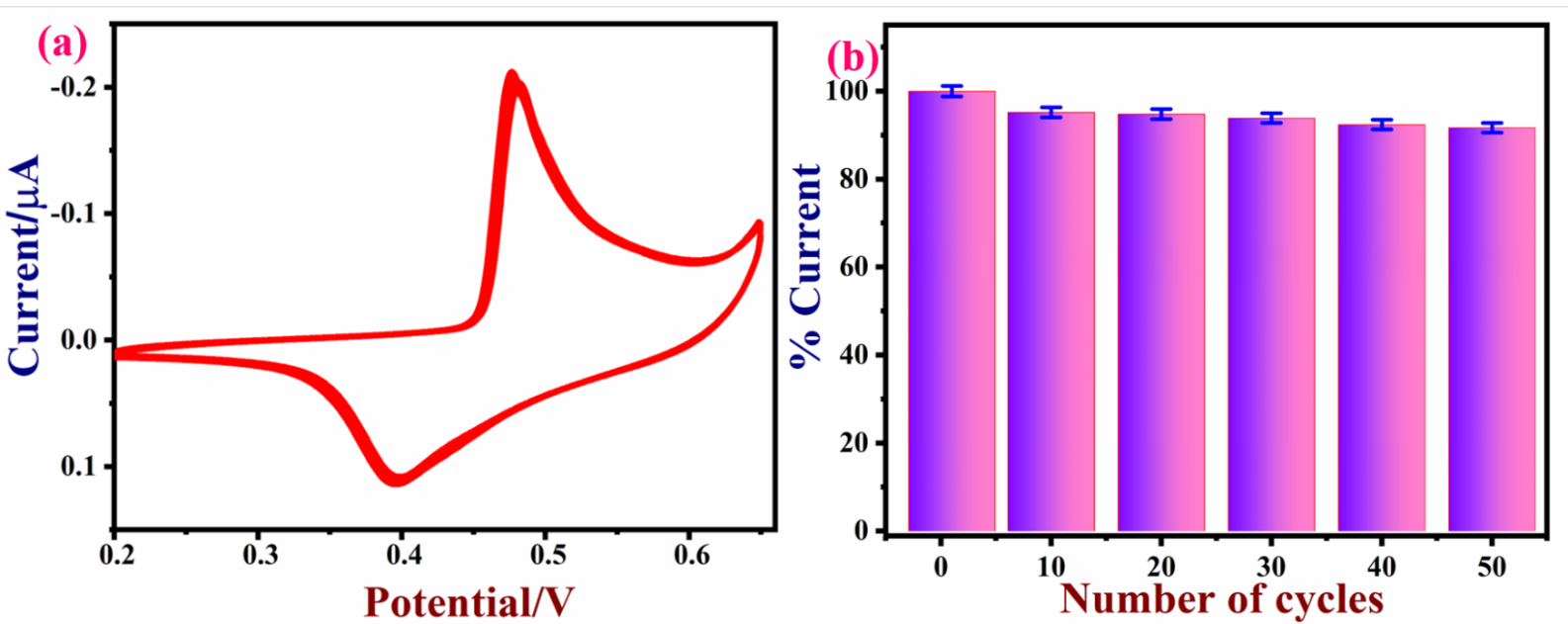


**Fig. S5** (a) CVs were recorded continuously for 50 cycles using a NiO/MoSe_2_/GCE in 0.1 M NaOH at a scan rate of 50 mVs^-1^. (b) Histogram represents changes in peak currents against number of potential cycles.

**

**

**Fig. S6** CVs were recorded using three independently prepared NiO/MoSe_2_ modified GCE’s for the electro-oxidation of 50 µM glucose: (Black curve: NiO/MoSe_2_/GCE-1, red curve: NiO/MoSe_2_/GCE-2, and blue curve: NiO/MoSe_2_/GCE-3).

**

**

**Fig.S7** The histogram was made between serum glucose oxidation currents and storage periods using the same NiO/MoSe_2_/GCE.
